# Virus-Like Particle Vaccine Protects against 2009 H1N1 Pandemic Influenza Virus in Mice

**DOI:** 10.1371/journal.pone.0009161

**Published:** 2010-02-11

**Authors:** Fu-Shi Quan, Aswani Vunnava, Richard W. Compans, Sang-Moo Kang

**Affiliations:** Department of Microbiology and Immunology and Emory Vaccine Center, Emory University School of Medicine, Atlanta, Georgia, United States of America; Federal University of São Paulo, Brazil

## Abstract

**Background:**

The 2009 influenza pandemic and shortages in vaccine supplies worldwide underscore the need for new approaches to develop more effective vaccines.

**Methodology/Principal Findings:**

We generated influenza virus-like particles (VLPs) containing proteins derived from the A/California/04/2009 virus, and tested their efficacy as a vaccine in mice. A single intramuscular vaccination with VLPs provided complete protection against lethal challenge with the A/California/04/2009 virus and partial protection against A/PR/8/1934 virus, an antigenically distant human isolate. VLP vaccination induced predominant IgG2a antibody responses, high hemagglutination inhibition (HAI) titers, and recall IgG and IgA antibody responses. HAI titers after VLP vaccination were equivalent to those observed after live virus infection. VLP immune sera also showed HAI responses against diverse geographic pandemic isolates. Notably, a low dose of VLPs could provide protection against lethal infection.

**Conclusion/Significance:**

This study demonstrates that VLP vaccination provides highly effective protection against the 2009 pandemic influenza virus. The results indicate that VLPs can be developed into an effective vaccine, which can be rapidly produced and avoid the need to isolate high growth reassortants for egg-based production.

## Introduction

Influenza is a serious human respiratory disease causing recurrent outbreaks, significantly affecting human health and the global economy. In April 2009, several human cases infected with a novel H1N1 swine-origin influenza virus A (SOIV) were reported in Mexico and in the United States [1]–[4]. This virus spread rapidly to over 74 countries around the world by early June 2009 when the WHO raised the global outbreak alert level to the pandemic phase 6 [3], [5]. WHO regional laboratories reported at least 12,220 confirmed deaths from the 2009-H1N1 pandemic influenza virus as of December 27, 2009 (http://www.who.int/csr/don). SOIV shows an unusually rapid rate of spread, emerging outside of the normal seasonal period for the virus [2].

Three previous influenza pandemics were caused by the A/H1N1 virus in 1918 to 1919, A/H2N2 from 1957 to 1963, and A/H3N2 from 1968 to 1970 [6]. These previous pandemics had distinct characteristics such as a shift to a new antigenic subtype of virus, higher mortality in younger populations, multiple pandemic waves, and higher transmissibility than seasonal influenza. Influenza A virus infects various host species including birds, swine, and humans. The new 2009 SOIV (H1N1) virus was found to contain a combination of gene segments that had not been previously identified in swine or human influenza isolates [7]–[9]. The HA, NP, and NS genes of the new 2009 pandemic strain were derived from classical swine virus and are closely related to the 1918 human pandemic virus. The NA and M genes are from a Eurasian swine virus. The PB2 and PA genes originated from an avian virus, and were introduced into the swine viruses. PB1 is similar to that of human H3N2 virus that acquired the PB1 gene from an avian virus. There is a concern that further mutation and/or acquisition of virulence genes derived from other human or animal influenza viruses could change the new pandemic strain into a more pathogenic one than it is now [10], [11].

Large-scale mass vaccination is the most effective measure to control the pandemic. However, due to extensive antigenic drift which occurred in the 2009 pandemic virus, current seasonal vaccines do not provide any significant cross protection [12]. The current approach using embryonated hen's eggs for large scale virus growth and vaccine manufacture is problematic. During some recent years, there have been shortfalls in vaccine supply in response to the influenza season. Local or systemic allergic reactions to residual egg proteins in the vaccine components can occur in some individuals. Significant shortages and delays happened in the supply of the 2009 pandemic vaccine, due in part to lower growth in egg substrates compared to those observed with seasonal vaccines. Developing an effective approach for vaccine production that does not rely on the egg supply is highly desirable particularly for pandemic viruses. Mammalian cell derived influenza vaccines were found to be immunogenic and can provide an alternative system for vaccine production [13], [14]. Nevertheless, these approaches still rely on growing live viruses for vaccine production [14]. In contrast, production of virus-like particles (VLPs) in insect cells can avoid the handling of live influenza viruses during the vaccine manufacturing process [15]. Also, influenza VLPs were shown to induce broader immune responses than egg-produced inactivated viral vaccines [16].

In this study, we have investigated the immunogenicity and protective efficacy of 2009 pandemic influenza VLPs after a single dose vaccination. Results on protective immune correlates and the breadth of protective immunity are presented.

## Materials and Methods

### Virus, Cells and Antibody

H1N1 influenza virus (A/California/04/2009) kindly provided by Dr. Richard Webby, A/New Caledonia/20/99 virus provided by Dr. Donald F. Smee, 2009 H1N1 reassortant viruses provided by Dr. Ruben Donis, and A/PR8/1934 were grown in 11-day old embryonated hen's eggs. Egg allantoic fluids were harvested and stored at −80°C until use. MDCK cells were maintained in Dulbecco's modified Eagle's medium (DMEM) and used to determine virus titers from egg allantoic fluids and mouse lung homogenates by plaque assay. Mice were infected with serial dilutions of A/California/04/2009 virus and the 50% lethal dose (LD_50_) was determined.

### Generation of Recombinant Baculovirus (rBV) Expressing HA and M1 of A/California/04/2009 (H1N1) Virus

A plasmid PCI containing cDNA encoding HA derived from influenza new H1N1 (A/California/04/2009) was kindly provided by Dr. Ruben Donis (CDC, Atlanta, GA). The HA gene was PCR amplified with primers containing flanking restriction enzyme sites for cloning into the pFastBac plasmid expression vector. (forward primer, 5- AAA GAATTC ACC ATG AAG GCA ATA CTA GTA G 3-; reverse primer, 5- TTA CTCGAG TTA AAT ACA TAT TCT ACA CTG 3-; EcoRI and XhoI sites are underlined). For M1 gene cloning, A/California/04/2009 virus was inoculated into MDCK cells and total viral RNA was extracted using an RNeasy Mini kit (Qiagen). Reverse transcription (RT) and PCR were performed on extracted viral RNA using the One-Step RT-PCR system (Invitrogen) with gene specific oligonucleotide primers. The following primer pairs were used for M1: 5- AAA GAATTC ACC ATG AGT CTT CTA ACC GAG GT 3-; and 5- TTA CTCGAG TTA CTC TAG CTC TAT GTT GAC-3. Following RT-PCR, a cDNA fragment containing the M1 gene was cloned into the pFastBac vector (Invitrogen). The nucleotide sequences of the HA and M1 genes were identical to the previously published sequences (accession numbers FJ966082 for HA, FJ966085 for M1). Recombinant baculoviruses (rBVs) expressing HA and M1 of A/California/04/2009 virus were generated as described previously [17].

### Preparation of Influenza VLPs

Sf9 insect cells were co-infected with recombinant BVs expressing HA and M1, and culture supernatants were harvested to purify VLPs as described [17]. Characterization of influenza VLPs was performed by silver staining of sodium dodecyl sulfate polyacrylamide gel electrophoresis (SDS-PAGE) as well as western blot using mouse polyclonal antibodies raised by live virus infection with the 2009 H1N1 pandemic virus (A/California/2009) as previously described [18]. HA contents in purified influenza VLPs were estimated by hemagglutination activity assay and western blot in comparison with inactivated A/California/2009 virus. Influenza VLPs were found to contain approximately 0.1 µg HA (A/California/2009) per 1 µg of total protein of VLPs (∼10%), which is a similar level as previously described for other influenza VLPs [19], [20]. For negative staining of VLPs, sucrose gradient-purified VLPs were applied to a carbon-coated formvar grid, and the grid was stained with 1% phosphotungstic acid.

### Immunization and Challenge

Female inbred BALB/c mice (Charles River) aged 6 to 8 weeks were used. Groups of mice (12 mice per group) were intramuscularly immunized with 10 or 0.1 µg (total protein) of VLPs. For challenge studies, naïve or vaccinated mice were isoflurane-anesthetized and intranasally infected with 100 or 10 LD_50_ of A/California/04/2009 or A/PR/8/1934 virus (10 LD_50_) in 50 µl of phosphate-buffered saline (PBS). Mice were observed daily to monitor changes in body weight and to record mortality (25% loss in body weight as the Institutional Animal Care and Use Committee (IACUC) endpoint). All animal experiments and husbandry involved in the studies presented in this manuscript were conducted under the guidelines of the Emory University IACUC. Emory IACUC operates under the federal Animal Welfare Law (administered by the USDA) and regulations of the Department of Health and Human Services.

### Antibody Responses and Hemagglutination Inhibition (HAI) Titer

Blood samples were collected by retro-orbital plexus puncture at week 1, 2 and 5 after immunization, and both sera and lung homogenates were obtained at day 4 after challenge infections. Influenza virus specific IgG, IgG1, IgG2a, and IgA antibodies were determined by enzyme-linked immunosorbent assay (ELISA) as described previously [18]. As coating antigens to measure virus specific antibodies, inactivated egg-grown viruses were coated onto 96-well microtiter plates. HAI titers were determined using 0.5% chicken red blood cells and 4 HA units per well of A/California/2009, A/PR8 or H1N1 reassortants.

### Assays of Lung Viral Titers, Cytokine, and Antibody Secreting Cells (ASC)

Lung samples, spleens, and bone marrow were collected at day 4 post challenge. Determination of viral titers in lung extracts was performed using MDCK cells as described [18]. Cytokine interferon (INF-γ) ELISA was performed as described previously [18]. Ready-Set-Go IFN-γ kits (eBioscience, San Diego, CA) were used for detecting cytokine levels in lung extracts following the manufacturer's procedure. For ASC assays, 96-well culture plates were coated with A/California/04/2009 or A/PR8 virus overnight, and spleen and bone marrow cells were added to coated plates after blocking. Secreted antibody levels were determined after 2 or 6 days in vitro culture.

### Statistics

All parameters were recorded for individuals within all groups. Statistical comparisons of data were carried out using the t-test of the SigmaPlot (Systat Software, Inc.). A P value less than 0.05 was considered to be significant.

## Results

### Characterization of A/California/04/2009 VLPs

**Figure 1:**
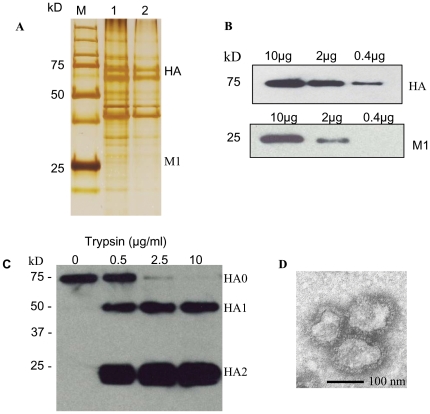
Silver stained SDS-PAGE, western blot and electron microscopy examination. (A) Silver stained gel showing HA and M1 bands in A/California/04/2009 H1 VLPs. M: a standard molecular size marker, Lane 1∶2.5 µg of purified influenza VLP protein, Lane 2∶1 µg of purified influenza VLP protein. (B) The incorporation of A/California/04//2009 H1N1 influenza HA or M1 into VLPs (10, 2, and 0.4 µg of total protein) was determined by Western blot using mouse anti-2009 H1N1 sera or anti-M1 IgG antibody. (C) Cleavage of A/California/04/2009 virus HA in VLPs. VLPs containing HA (10 µg of total protein) were incubated for 5 min at 37°C with different concentrations of TPCK treated trypsin, resolved by SDS-PAGE, and probed by Western blotting. The thicker bands of the HA2 subunit are commonly observed after trypsin treatment due to the more effective transfer of HA2 during western blot. Lanes from left to right represent 0, 0.5, 2.5 and 10 µg/ml trypsin respectively. (D) Electron microscopy of influenza H1N1 VLPs.

We produced 2009 SOIV VLPs in insect cells co-infected with recombinant baculoviruses (rBVs) expressing the M1 matrix and HA glycoprotein derived from A/California/04/09 (H1N1) virus following a procedure previously described [17]. The incorporation of HA and M1 into VLPs was confirmed by silver-stained SDS-PAGE (Fig. 1A) and western blot using immune sera obtained from mice infected with the A/California/2009 virus (Fig. 1B). HA was found to be one of the dominant proteins in VLPs (Fig. 1A). HA incorporated into VLPs was found to be predominantly in the precursor form, and was found to be cleaved into HA1 and HA2 subunits by trypsin treatment (Fig. 1C). The hemagglutination activity of the VLPs (1 mg protein/ml) was found to have approximately 2,560 HA titers, which indicates the functional integrity of HA incorporated into VLPs. The size and morphology of the 2009 H1 VLPs resemble influenza virus particles, with spikes on their surfaces characteristic of influenza virus HA proteins on virions (Fig. 1D). Taken together, these results show that H1 VLPs produced in insect cells contained HA with functional activity and were structurally intact, resembling influenza virions in morphology and size.

### A Single Immunization with 2009 H1 VLPs Elicits Antibody and HAI Responses

**Figure 2:**
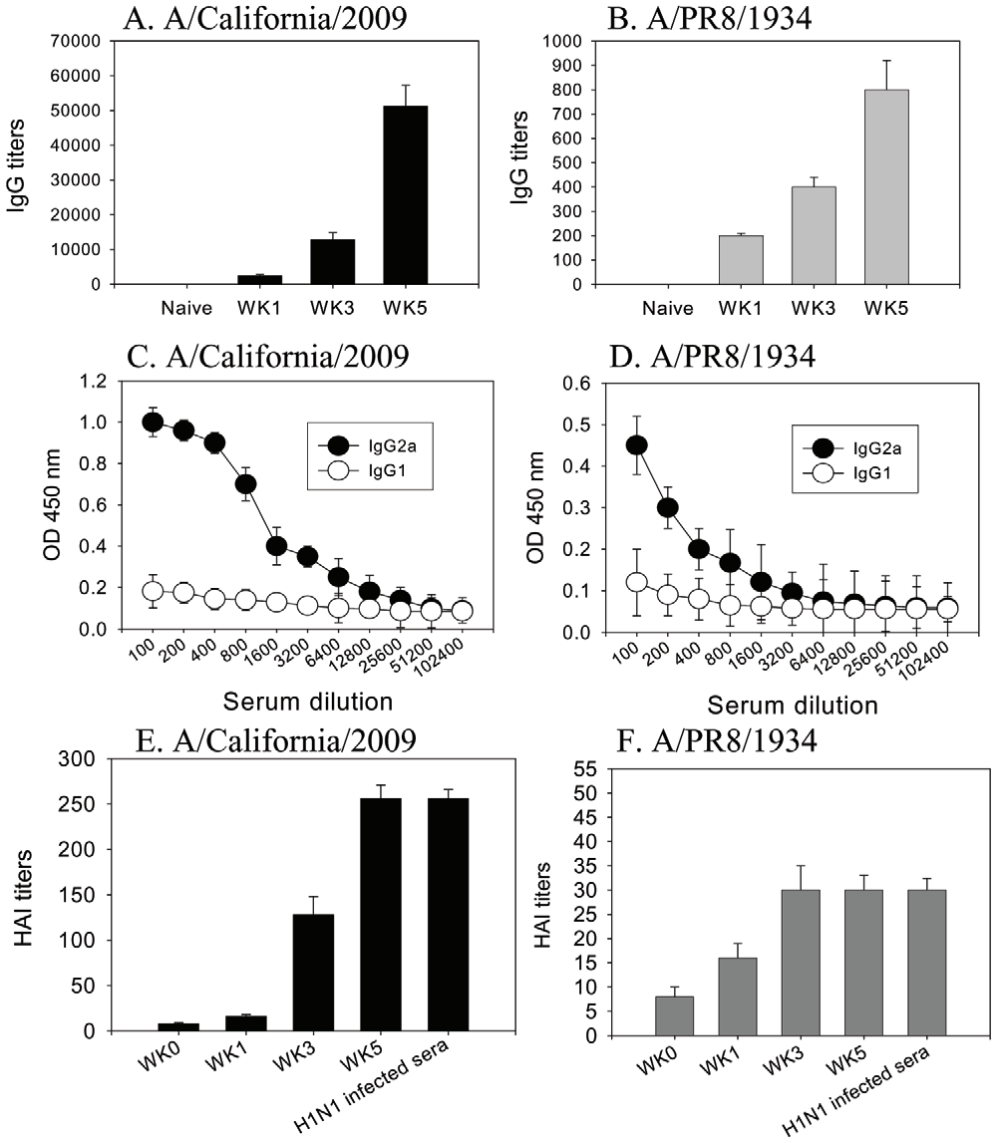
Humoral responses. A–B: IgG serum antibodies specific to A/California/04/2009 (A) or A/PR8/34 (B) H1N1 influenza virus were determined at week 1, 3, 5 in the group of mice that were intramuscularly immunized with 10 µg of VLPs. Titers are expressed as the highest dilution of serum having a mean optical density at 450 nm greater than the mean plus 2 standard deviations above naive serum samples. Significantly higher IgG titers against A/California/04/2009 or PR8 viruses were detected at week 3 compared to week 1 (P<0.01); and at week 5 compared to week 3 (P<0.001). C–D: IgG2a and IgG1 responses. Serum was serially diluted and ELISA was performed for serum antibodies specific to A/California/04/2009 (C) or PR8 viruses (D). E–F: HAI titers. HAI titers against A/California/04/2009 (E) or A/PR8/34 (F) viruses at week 0, 1, 3 and 5 after a single immunization were determined. A/California/04/2009 infected sera at 5 weeks after infection were used as control. Significant HAI titers against A/California/04/2009 viruses were determined at week 5 compared to week 3 or week 1 (P<0.01). Significant HAI titers against A/PR8 viruses were also determined at week 5 compared to week 1 (P<0.01).

To evaluate VLP immunogenicity, groups of mice (n = 12) were immunized intramuscularly with 10 µg of VLPs (approximately 1 µg HA). We determined the levels of total IgG antibody responses specific to the A/California/04/2009 and cross reactive to the antigenically different A/PR/8/1934 virus (A/PR8) (Fig. 2AB) at 1, 3, and 5 weeks after a single immunization with VLPs. IgG responses specific to the A/California/04/2009 virus and cross reactive to the PR8 virus increased with time post immunization (P<0.01), indicating the progressive maturation of virus-specific antibodies. Even with a low dose of VLP (0.1 µg), a similar pattern of antibody levels that increased up to 5 weeks after vaccination was observed (Table 1). Although the difference was 100 fold between high (10 µg) and low (0.1 µg) VLP vaccine doses, the antibody titers showed only around a 3 fold difference (Fig. 2, Table 1). As expected, mice immunized with VLPs induced significantly higher levels of IgG antibodies specific to the homologous virus by over 60 fold, compared to A/PR8 virus, which indicates that these two strains are distantly related in terms of antigenic properties.

**Table 1:** IgG antibody responses with low dose (0.1 µg) of VLPs.

Naïve	week 1	week 3	week 5
150±22	400±50	6400±780	19200±2300

IgG serum antibodies specific to A/California/04/2009 influenza virus were determined at week 1, 3, 5 in the group of mice immunized with low dose 1 µg of VLPs. Titers are expressed as the highest dilution of serum having a mean optical density at 450 nm greater than the mean plus 2 standard deviations of naive serum samples. Significant higher IgG titers against new H1N1 were detected at week 3 compared to week 1 (P<0.01); and at week 5 compared to week 3 (P<0.001).

IgG2a dominant antibody responses specific to the A/California/2009 virus were observed in immune sera (Fig. 2C). Also, significant levels of IgG2a antibodies cross-reactive to A/PR8/1934 virus were observed at lower levels in VLP immune sera (Fig. 2D). Taken together with results including total IgG and the pattern of isotypes induced after a single vaccination, VLPs are highly immunogenic and can induce virus specific antibody responses with some cross reactivities.

To investigate immune correlates for predicting protection, we determined hemagglutination inhibition (HAI) titers in immune sera collected 1, 3, and 5 weeks after immunization (Fig. 2EF). Consistent with levels of the pandemic virus-specific antibodies, the immune sera showed progressive increases in HAI titers up to 250 against the homologous A/California/2009 strain. Importantly, the HAI titers induced by a single dose of VLP vaccination were comparable to those obtained by live virus infection with A/California/2009 (Fig. 2E). As expected, the HAI titers against A/PR8 (Fig. 2F) were significantly lower by over 8 fold compared to those against the homologous strain A/California/2009, suggesting that the immune responses preferentially recognize the homologous vaccine strain. Cross reactive HAI activities were also determined against seasonal influenza H1N1 virus, A/New Caledonia/20/1999. The HAI titers were 8–16 with immune sera collected at weeks 1–5 after vaccination, indicating that cross-reactive HAI titers against seasonal influenza virus are close to the background levels.

Interestingly, the VLP vaccine immune sera showed high HAI titers against reassortant viruses containing HA and NA derived from pandemic isolates from different geographical sites including Texas and New York (Table 2), indicating that these isolates are closely related antigenically. These results indicate that the VLP vaccines can induce protective functional antibodies to variant 2009 H1N1 isolates at high levels and to an antigenically distant strain at significantly lower levels.

**Table 2:** HAI titers against 2009 H1N1 isolates.

Serum	Virus strain			
	^1^A/California/04/2009	^2^RG A/Texas/5/2009	^2^RG A/Texas/5/2009*	^2^RG A/New York/18/2009
Immune sera	256±28	480±35	720±49	480±34
Infected sera	256±9	480±24	720±38	480±25
Naïve sera	8±0.5	16±4	16±4	16±2

HAI titers against different strains of new H1N1 viruses were determined using immune sera collected from mice at week 5 after immunization with A/California/04/2009 VLP vaccine (immune sera), from mice infected with A/California/04/2009 (infected sera), or naïve sera. ^1^Wild type A/California/04/2009 virus. ^2^Three reassortant viruses, kindly provided by Dr. Ruben Donis (CDC, Atlanta, GA), were generated with six A/PR/8/34 internal genes and with HA and NA of A/Texas/5/2009, A/Texas/5/2009* (Q226R mutation in HA), A/New York/18/2009 respectively. Viruses were grown in eggs and used for HAI titers using 4 HA units.

### Protection against Lethal Challenge

To determine the efficacy of a lower vaccine dose, additional groups of mice were immunized intramuscularly with 0.1 µg VLPs (approximately 0.01 µg HA) once or twice (weeks 0 and 4), and then challenged with a lethal dose (10 LD_50_) of the homologous virus (A/California/04/2009) at 10 days after the last immunization. Since levels of antibody responses were relatively low in the 0.1 µg VLP immunized mice, a lethal dose of 10 LD_50_ was used for challenge studies, which is still high enough for testing vaccine efficacy [18]. Both groups of mice immunized with 0.1 µg VLPs were protected against lethal challenge (Table 3). The group with two immunizations displayed no loss in body weight, whereas the single low dose group exhibited moderate loss in body weight. Therefore, a dose as low as 0.1 µg of VLPs can provide protection against lethal infection at as early as 10 days post vaccination even with a single dose.

**Table 3:** Protection of mice immunized with a low dose of VLPs.

Group	Body weight changes (%)								Survival (%)
	D0	D3	D4	D6	D8	D10	D12	D14	
Two immunization	100	100	100	100	100	100	100	100	100
Single immunization	100	92	89	88	93	97	98	99	100
Naïve	100	91	87	75					0

Mice were intramuscularly immunized with 0.1 µg of VLPs once or twice, and were challenged with a lethal dose of A/California/04/2009 (10 LD_50_) (n = 6) day 10 post immunization. Mice were observed daily to monitor changes in body weight and to record mortality (25% loss in body weight as the IACUC endpoint).

**Figure 3:**
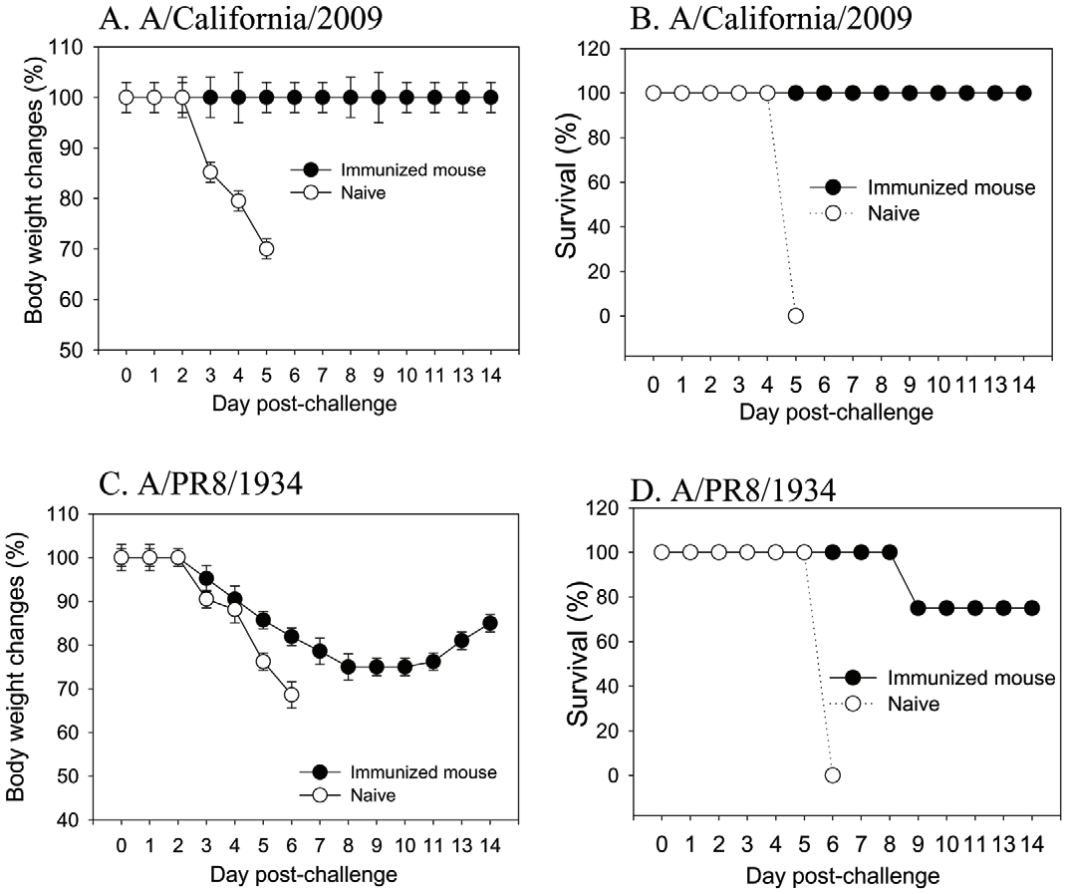
Protection of mice from lethal influenza virus challenge. A–B: Protection against A/California/04/2009 virus challenge. Mice intramuscularly immunized with a single dose of VLPs (10 µg) were challenged with a lethal dose (100 LD_50_) of A/California/04/2009 virus at week 6 post immunization. Mice (n = 12) were monitored daily for 14 days for body weight changes (A) and survival rates (B). C–D: Protection against the antigenically distant A/PR8/1934 virus (10 LD_50_). Body weight changes (C) and survival rates (D) are shown.

To determine the potency of protective efficacies, mice were challenged with a high lethal dose of A/California/2009 virus (100 LD_50_) at 6 weeks after a single immunization with VLPs. As shown in Fig. 3, all naïve mice died after infection with the wild type A/California/2009 virus. In contrast, vaccinated mice were completely protected when challenged with the homologous A/California/2009 virus and did not show any loss in body weight (Fig. 3AB). A similar protective efficacy was observed 4 months after VLP vaccination (data not shown). To determine the potential cross protection against an antigenically distant strain, immunized mice were also challenged with A/PR8 virus (10 LD_50_). VLP immunized mice showed a significant level of protection, with 75% survival rates against A/PR8 virus, although the surviving mice exhibited approximately 20% transient loss in body weight (Fig. 3CD).

**Figure 4:**
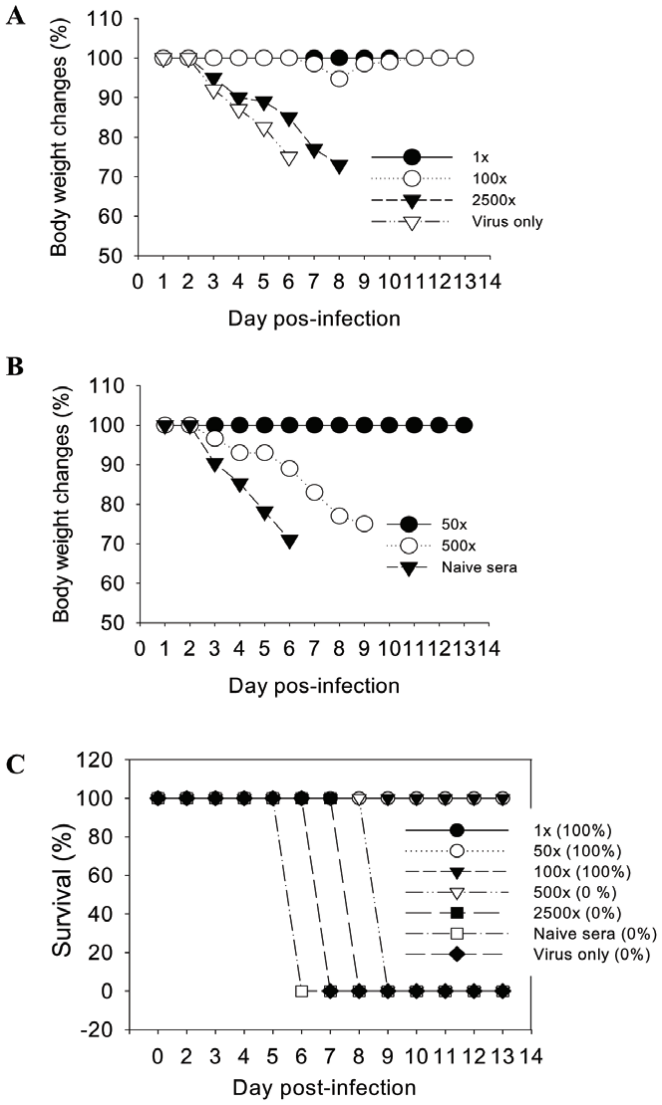
Protective role of immune sera. Naive sera from unimmunized mice or immune sera from vaccinated mice (10 µg VLP single dose) were serially diluted (1X, 50X, 100X, 500X, and 2500X or naïve sera). These diluted serum samples (20 µl) were mixed with 40 µl of A/California/04/2009 virus (10 LD_50_) and incubated for 30 min at 30°C. Mice (n = 4 BALB/c mice per each diluted serum-virus group) were intranasally infected with an in vitro incubated mixture of naïve or immune sera and A/California/04/2009 virus (10 LD_50_), and monitored daily for 14 days for body weight changes (A, B). Survival rates (C). The numbers in the parenthesis indicate survival rates in each infected group.

The role of immune sera in providing protection was evaluated in mice that received a lethal dose of virus mixed with immune or naïve sera (Fig. 4AB). Immune sera at dilutions up to 100 fold conferred protection with only a transient body weight loss whereas a 50 fold dilution provided protection without any loss in body weight. Higher dilutions of immune sera did not give any protection although body weight loss was delayed compared to the naïve serum control. These results suggest an important role of humoral responses in providing protection.

Overall, these results indicate that a single low dose of VLPs can confer protective immunity against lethal challenge with the new pandemic virus. Also, influenza VLP vaccines provide some cross protection against an antigenically distant strain in the mouse model.

### VLP Vaccination Provides Effective Control of Challenge Virus Replication

**Figure 5:**
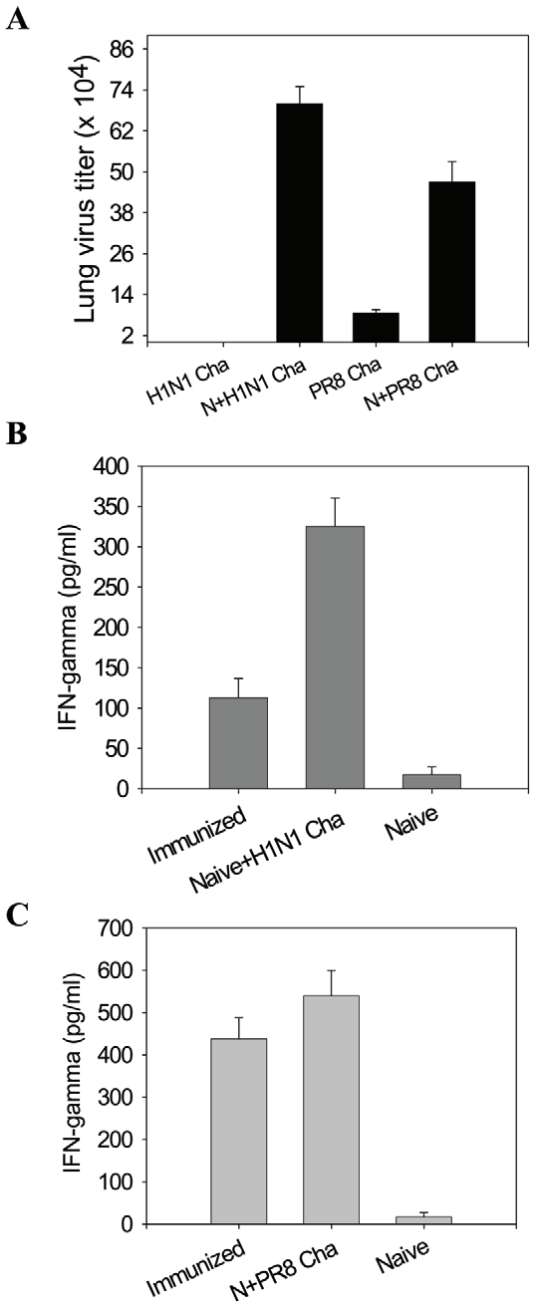
Lung virus titer and inflammatory cytokine IFN-gamma. (A) Lung virus titers. Lung samples from individual mice immunized with 10 µg VLPs in each group (n = 6) were collected on day 4 post-challenge with a lethal dose of A/California/04/2009 or A/PR8/1934 virus. Each lung sample from a mouse was suspended in 1 ml with Dulbecco's modified Eagle's medium. Statistical significance is indicated between groups of mice challenged with A/California/04/2009 (P<0.001) or A/PR8/34 (P<0.01) compared to naive mice challenged with the same lethal dose. (B) Lung inflammatory cytokine IFN-γ after A/California/04/2009 challenge. (C) Lung inflammatory cytokine IFN-gamma after A/PR8/1934 challenge. H1N1 Cha, VLP immunized mice after A/California/04/2009 challenge, N+H1N1 Cha: Naïve mice after A/California/04/2009 challenge, PR8 Cha: VLP immunized mice after A/PR8/1934 challenge, N+PR8 cha: Naive mice after A/PR8/1934 challenge. Naïve: Untreated mice.

The efficiency of virus clearance in lungs provides a sensitive indicator for assessing protective efficacy. At day 4 post challenge, mice were sacrificed and viral titers in lung extracts were determined (Fig. 5A). The naïve mouse control groups showed high lung viral titers. In contrast, in mice immunized with VLPs, A/California/2009 viral titers were below the detection limit (50 pfu per lung). When the VLP immunized mice were challenged with A/PR8/1934 virus, a five fold reduction in lung titers was observed in the vaccinated mice compared to those in the naïve controls (Fig. 5A).

The H1N1 A/California/2009 virus was found to be lethal to mice without adaptation although the pathogenesis of this virus remains largely unknown. To determine whether vaccination would diminish the production of inflammatory cytokines in lungs, we determined the levels of interferon γ (INF-γ) in lung extracts collected at day 4 post lethal challenge infection. Naïve mice infected with A/California/2009 virus showed high levels of IFN-γ (over 300 pg/ml) in lung extracts and eventually all died (Fig. 5B). The vaccinated mice exhibited 3 fold lower levels (approximately 100 pg/ml lung extracts) of IFN-γ compared to those observed in the naïve infected control (over 300 pg/ml) but significantly higher than the uninfected naïve control, indicating that viral replication occurred prior to clearance. As a comparison, the A/PR8 infected naive (unvaccinated) mice exhibited 550 pg IFN-γ per ml, and VLP vaccinated mice were found to have a little decrease in IFN-γ levels after A/PR8 infection (Fig. 5C). Therefore, these results indicate that VLP vaccination can confer effective control of viral replication, resulting in reduced proinflammatory cytokine production.

### VLP Vaccination Induces Effective Recall Immune Responses

**Figure 6:**
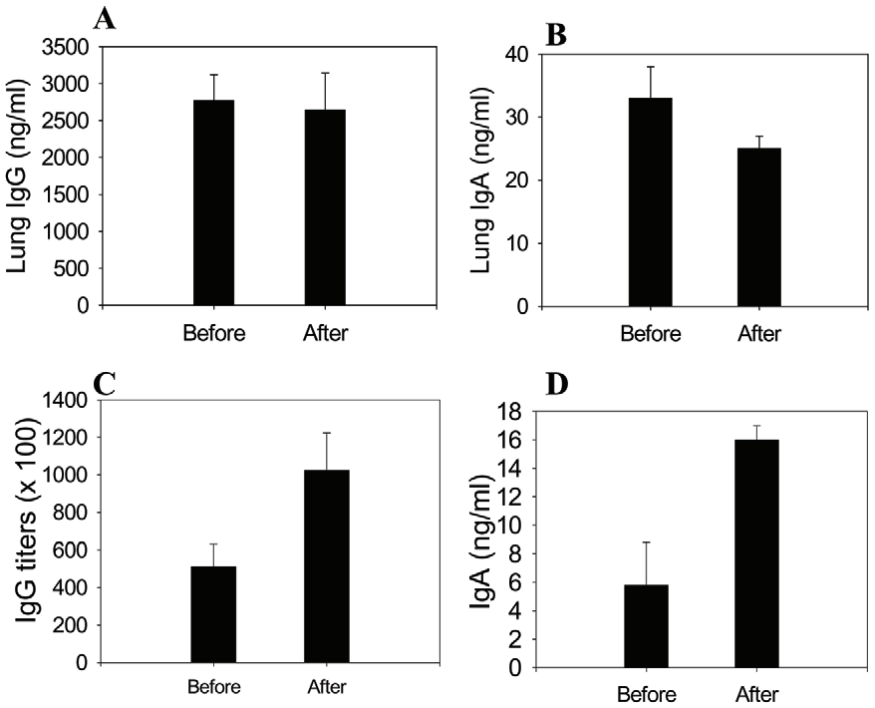
Recall antibody responses in lung and serum. Lung IgG (A) and IgA (B), and serum IgG (C) and IgA (D) antibody responses to A/California/04/2009 virus were determined before (week 6.5 post immunization) and after challenge (day 4 post challenge) with the homologous virus A/California/04/2009. Lung and serum samples before and after challenge were collected at the same time (n = 6) and analyzed under the same assay condition (week 6.5 post-vaccination). Lung IgA (B) before and after challenge: P<0.05. Serum IgG (C) and IgA (D) responses before and after challenge from the A/California/04/2009 virus challenge: P<0.001. Low and moderate naïve backgrounds were observed in the serum and lung samples respectively and these values have been subtracted from the immune samples.

A goal of vaccination is to confer the host with immunity to respond rapidly upon encounter with a pathogen. As a measure of recall immune responses, we compared the immune responses before and after challenge infection. Virus specific antibody responses over background were not found in lungs and sera of naïve mice at 4 day post challenge with A/California/2009 virus (data not shown). VLP vaccinated mice showed high levels of lung IgG antibodies specific to the homologous virus, and their levels were similar before and after challenge (Fig. 6A). Lower levels of lung IgA antibodies were observed after challenge compared to those before challenge (Fig. 6B, P<0.05 before and after challenge).

In contrast to lung antibodies, significant higher levels of serum IgG and IgA antibody responses were detected at day 4 post challenge with the homologous A/California/04/2009 (Fig. 6C and 6D) compared to those before challenge. Serum IgG antibody levels specific to the homologous virus were 60 and 15 fold higher than those specific to the antigenically different A/PR/8/1934 virus at the time of before and after challenge respectively (data not shown). Nonetheless, it is interesting to note that IgA antibodies were found to be induced in sera of mice systemically vaccinated with VLPs. Overall, these results indicate that VLP vaccination can confer effective recall immune responses, which are likely to contribute to protective immunity.

**Figure 7:**
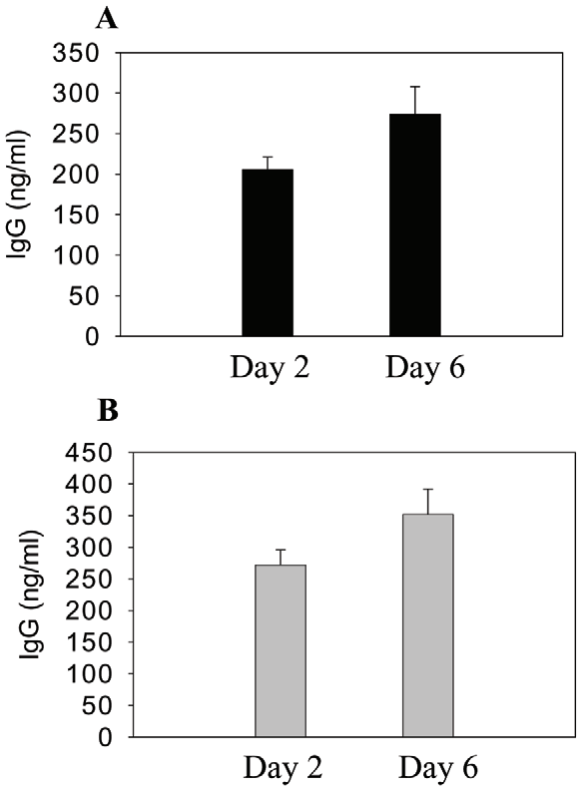
Antibody secreting cells (ASC). Antibody secreting cells (ASC) from spleen and bone marrow. A: Mouse spleen monolayer cells were prepared at day 4 post challenge. ASC were determined after 2 or 6 days of in vitro culture specific to A/California/04/2009 virus. B: Antibody secreting cells (ASC) from bone marrow. Cells from mouse bone marrow at day 4 post challenge were prepared in vitro. ASC for IgG was determined after cultured in vitro for 2 or 6 days to A/California/04/2009 virus.

To determine the antibody secreting cell responses, spleen cells were harvested at day 4 post challenge infection and subjected to in vitro culture. After 2-day' cultures, high levels of antibodies specific to A/California/2009 viral antigens were found in culture supernatants of spleen cells from mice vaccinated with VLPs but not from unvaccinated mice (Fig. 7A). Antibodies specific to A/PR8/1934 viral antigen were also found to be secreted at low levels after 6 days of in vitro culture (data not shown).

When we analyzed antibody secreting cell responses in the bone marrow where long-lived plasma cells reside, high levels of A/California/2009-specific antibodies were found to be secreted into bone marrow culture supernatants (Fig. 7B). Similar levels of antibody secreting cell responses were observed before challenge (data not shown). In summary, these results suggest that a single vaccination with VLP vaccines can induce antibody secreting plasma cell responses at an early time point post challenge infection. Thus, VLP vaccination can induce memory B cells that can rapidly differentiate into antibody secreting plasma cells upon exposure to a pathogen.

## Discussion

The ongoing, rapidly spreading influenza pandemic to which the human population has little immunity is a great public health concern particularly for children and young adults. Intramuscular vaccination is the common delivery route for most vaccines including influenza. In the present study, we tested the immunogenicity and protective efficacy of pandemic VLPs after a single dose intramuscular vaccination. We found that vaccinated mice were completely protected against challenge infection with a high lethal dose of the A/California/2009 virus, and that viral replication in the lung was reduced to levels below the detection limit. The insect cell expression system provides an alternative approach for scaling-up mass production of vaccines. VLPs containing biologically active glycoproteins from different influenza subtypes have been previously produced in insect cells and have been shown to elicit strong immune responses conferring protection against homologous or related heterologous viruses [18], [21], [22].

Most previous studies have focused on immune responses and protection induced after a prime-boost immunization regimen. It is significant, as demonstrated in this study, that a single intramuscular dose of VLPs can provide complete protection against a high lethal dose (100 LD_50_) of wild type A/California/2009 virus with no detectable viral titers in the lung, the major site for viral replication. In a dose sparing test, a very low dose of VLPs (∼0.01 µg HA) was also found to provide protection against lethal infection as early as 10 days post vaccination. Even at week 5 post single vaccination with a low dose when antibody levels were higher than those at day 10 (Table 1), these mice still showed a moderate loss in body weight (data not shown). The boost vaccination has significantly improved protection efficacy without showing any body weight loss. These results indicate that VLPs are an attractive vaccine platform, possibly because of their particulate nature as well as the presentation of functional glycoproteins in a native conformation. Also, there is a high possibility that suspended culture of insect cells is relatively easy to be expanded to a large fermentation reactor scale with a competitive production cost.

Previous studies demonstrated that intranasal immunization with inactivated whole viral vaccines could induce heterosubtypic immunity in the presence of heat-labile enterotoxin or cholera toxin adjuvants using a prime-boost vaccination regimen [23]–[25]. However, there are concerns about potential adverse effects regarding the use of endotoxin adjuvants [26], [27]. A recent study has shown that two intramuscular immunizations with chimeric influenza VLPs containing a membrane-bound form of bacterial flagellin induced partial protection against a heterosubtypic virus challenge [28]. Induction of HA specific antibodies as well as high HAI titers is likely a major contributor to protection and effective clearance of virus, which was shown by the protective role of antibodies induced by VLPs. The same immune sera also showed low HAI titers cross reactive to A/PR8 virus, a 1934 isolate. The amino acid sequence homology between A/California/2009 and A/PR8/1934 is only 74.3% in the HA1 subunit, which is the major site determining antigenicity. Therefore, the present study demonstrating that a single intramuscular immunization with VLP vaccine in the absence of adjuvant can induce complete protection against the homologous virus, as well as partial protection against a distantly related strain, has significant implications for future vaccine strategies to overcome antigenic variation.

Due to the sequence variation between the 2009 pandemic and antigenically distant A/PR/8/1034 or seasonal influenza virus A/New Caledonia/20/1999, levels of cross reactive HAI titers to these viruses were relatively very low, which might not be protective. Thus, other immune factors might have contributed to the partial cross protective immunity, which may include the IgG2a and IgA antibody responses as well as T cell immune responses although this remains to be determined. The dominant IgG2a isotype antibody responses and the induction of rapid recall antibody-secreting cells are likely to play a role in the effective viral clearance and homologous protection as well as partial cross protection by the VLP vaccination. IgG2a isotype antibody is known to be more effective than other isotypes in clearing virus infection via multiple mechanisms including complement activation, stimulation of antibody-dependent cellular cytotoxicity and clearance of opsonized virus by macrophages [29]–[31]. Vaccinated mice showed high antibody levels in sera at day 4 post challenge, which is consistent with the generation of antibody secreting cells in spleen and bone marrow.

In conclusion, this study demonstrates highly effective immunity to the new pandemic virus by VLP vaccination, resulting in effective viral clearance and protection. VLP vaccines have advantages in vaccine production, not requiring fertilized egg substrates. Therefore, development of influenza VLP vaccines should have a significant impact on control of influenza.
